# Effect of formaldehyde exposure on phytochemical content and functional activity of *Agaricus bisporus* (Lge.) Sing.

**DOI:** 10.1007/s11356-024-33625-y

**Published:** 2024-05-11

**Authors:** Fatih Kutluer

**Affiliations:** Department of Herbal and Animal Production, Kırıkkale Vocational School, Kırıkkale University, Kırıkkale, Turkey

**Keywords:** *Agaricus bisporus*, Antioxidant activity, Formaldehyde, Phenolic content

## Abstract

In this study, the effect of formaldehyde on phytochemical content and antioxidant activity of *Agaricus bisporus* was investigated. Synthetic compost based on wheat straw was prepared by fermentation and disinfection. After steam pasteurization, 5 g of *A. bisporus* mycelia were inoculated into 1 kg of compost. To determine the effects of formaldehyde, 2, 4, and 6% concentrations were added to the composts, while compost without formaldehyde was used for the control group. The harvesting period was set at 10 weeks. Total phenolic and flavonoid content, macro- and microelement profile, and phenolic content were analyzed in the harvested *A. bisporus* samples. Macro- and microelement content was determined by ICP-OES, and phenolic compound profile was determined by LC-MS/MS analysis. Formaldehyde levels in *A. bisporus* samples were determined by the acetylacetone spectrophotometry method. The antioxidant capacity of *A. bisporus* samples was determined by DPPH scavenging activity; antimutagenic effects of samples were determined by *Allium* test. Application of 2, 4, and 6% formaldehyde resulted in a 1.12-, 1.19-, and 2.07-fold reduction in total phenolic content, respectively. The total phenolic content was reduced between 34.4% and 71.8%. These changes were confirmed by LC-MS/MS analysis. Compounds such as protocatechuic acid, salicylic acid, ferulic acid, and 4-OH benzoic acid, which were detected in the control group, could not be detected in the samples treated with 6% formaldehyde, and it was found that the application of formaldehyde reduced the phenolic content. Similar changes were also observed in macro- and microelements, and significant changes in elemental contents were observed after formaldehyde application. While the presence of formaldehyde at a low level, which may be due to natural production, was detected in the control group, a residue of 11.41 ± 0.93 mg/kg was determined in the 6% FMD applied group. All these changes resulted in a decrease in the antioxidant activity of *A. bisporus*. The DPPH scavenging activity, which was determined in the range of 21.6–73.3% in the control samples, decreased to 12.3–56.7% in the samples treated with formaldehyde. These results indicate that the application of formaldehyde at different stages of *A. bisporus* cultivation leads to significant changes in the nutritional value and biological activity of *A. bisporus*.

## Introduction

The cultivation of edible mushrooms dates back to ancient times, and the cultivation of mushrooms, which began in France in the sixteenth century, started in Turkey in the 1970s, and developed rapidly. Today, modern production facilities have been established in Turkey in many different centers. The production of cultivated mushrooms in Turkey amounted to around 45,000 tons, and the annual per capita consumption of mushrooms was 579.2 grams in 2014 (Kutluer 2024). Although the number of edible mushrooms found in nature is quite high, the types of mushrooms that can be grown and produced are very limited. The most produced and consumed mushroom in the world is *Agaricus bisporus* (Lge.) Sing*.*, commonly known as the cultivated mushroom, and is an edible basidiomycete mushroom (Cappelli 1984). Edible mushrooms are species that grow spontaneously in nature and that humans learn about and consume through interaction with them. As a result of the study of edible mushrooms, their biological properties were determined, and cultivated mushrooms came into existence. Mushrooms are considered valuable, healthy food, because they contain few calories, fat, and essential fatty acids, but are rich in protein, vitamins, and minerals (Agrahar-Murugkar and Subbulakshmi 2005). In addition, medicinal properties such as antitumor, immunomodulatory, antimicrobial, antiplatelet, cholesterol-regulating, and blood sugar-lowering effects of *A. bisporus* have been reported (Reis et al. 2012a). These biological and pharmacological effects are related to the active substances it contains. Mushrooms are healthy foods for humans with high protein value and are also rich in vitamins B, C, D, and folic acid. *A. bisporus* (100 g) provide 22 kilocalories of energy and are an excellent source of riboflavin, niacin, and pantothenic acid as well as a good source of the mineral phosphorus. Most of the biological effects of *A. bisporus* are attributed to bioactive ingredients with antioxidant activity, such as phenolic compounds (Ferreira et al. 2009; Barros et al. 2009). Reis et al. (2012b) reported that *A. bisporus* contains high amounts of gallic acid as well as p-coumaric acid and protocatechuic acid in its phenolic profile. Mattila et al. (2001) found that *A. bisporus* contains various types of phenolic substances and the highest phenolic substance (2.69 μg/g) is cinnamic acid. They also reported that *A. bisporus*, an important source of mineral elements, contains high amounts of Se (3.2 mg/kg dry weight).

The main problems in mushroom cultivation are the inability to produce healthy, productive and marketable products. In order to prevent these problems and make mushroom cultivation more efficient, hygienic conditions must be provided. In addition, production materials must be healthy, and contamination with disease-causing and harmful microorganisms must be avoided. In particular, microbial contamination is one of the most important factors that cause product loss in mushroom cultivation, thus causing both economic and time loss (Claeys et al. 2009; Öztürk et al. 2017). Formaldehyde is used in the sterilization of incubation rooms, production rooms and cover soil. It is also used in fumigation and cleaning of post-production areas and in various stages of mushroom cultivation to prevent microbial contamination (Aksu et al. 1996). Formaldehyde exhibits a bactericidal effect on bacteria during their initial growth period and is used as a powerful sterilization and disinfection agent for this purpose. However, when used in high amounts, formaldehyde accumulates in mushroom tissues and can reach other organisms through the food chain. Liu et al. (2005) detected a high amount of formaldehyde (119–494 μg/g wet weight) in Shiitake mushrooms, which is harmful to human health. Shao et al. (2023) detected formaldehyde in 158 (76.33%) of 207 fresh mushroom samples, including *Agaricus bisporus*, collected from local markets in China, and found that the samples contained formaldehyde in the range of 90.2–8.90 mg/kg. Pesticides, disinfectants, and many xenobiotics that contaminate foods have serious toxic effects on living things. These chemicals cause toxicity in many plants and nutritional foods, especially by reaching through the soil, and cause genotoxic effects by contaminating humans (Yalçın et al. 2019; Tümer et al. 2022; Doğan et al. 2022; Çavuşoğlu and Yalçın 2023). Contaminate residues found in foods reach other organisms through the food chain and cause serious toxic effects. Since formaldehyde is highly reactive, it leads to cross-links with biological macromolecules and to formaldehyde-DNA-protein and protein-protein bonds. In this way, it interferes with numerous biochemical and physiological reactions in organisms (Claeys et al. 2009). Studies in the literature have shown that formaldehyde causes hepatotoxicity, tumor formation in the nasal epithelium, and various genotoxic effects (McGregor et al. 2006; Yalçın et al. 2015; Taşlı et al. 2015). Formaldehyde also exhibits physiological and biochemical effects on plants. In *Arabidopsis thaliana*, formaldehyde exposure has been reported to cause a significant increase in H_2_O_2_ content, increase in protein carbonyl levels and DNA-protein cross-links, and changes in anthocyanin content (Wang et al. 2012). Although there are studies that report formaldehyde residues in mushroom tissue, there is no study that investigates the effects of formaldehyde on the nutritional value of cultivated mushrooms. In this study, the effects of formaldehyde exposure on phytochemical content and antioxidant activity of *Agaricus bisporus* were investigated. The effect of formaldehyde on phenolic acid profile of *A. bisporus* was determined by LC-MC/MS analysis. The changes in macro- and micromolecules after formaldehyde application were studied by ICP-OES analysis. Formaldehyde levels in *A. bisporus* samples were determined by the acetylacetone spectrophotometry. The change in antioxidant and antimutagenic activity of *A. bisporus* was also investigated and associated with phenolic content.

## Material and methods

### Composting and cultivation

*A. bisporus* mycelia used in the study were obtained from the Kırıkkale University-Kirikkale Vocational School Mushroom Cultivation Programme. *A. bisporus* mycelia were propagated by subcultivation on potato dextrose agar culture medium. Wheat stalks were used as the main material for cultivation, and the compost formulation was based on the amounts in the standard compost formulation, which is accepted worldwide. Compost formulation for cultivation was prepared according to Baysal (2004). After steam pasteurization, when the compost temperature had decreased to 20–25 °C, 5 g of *A. bisporus* mycelia were inoculated into 1 kg of compost. To determine the effects of formaldehyde (FMD), 2, 4, and 6% FMD were added to the composts, while compost without FMD was used for the control group. On the day the mycelia were inoculated, formaldehyde was applied to the compost approximately 5 h. before. The harvest period was set at 10 weeks.

## Determination of formaldehyde levels

Formaldehyde levels in *A. bisporus* samples were determined by the acetylacetone spectrophotometry method. A 5 g of ground sample was mixed with 10 mL double-distilled water (ddH_2_O) at 4 °C for 30 min and homogenized at 5000 rpm for 15 min. Homogenate and 10% phosphoric acid solution were placed in the distiller pot. The receiving vessel was placed on ice, and distillation was performed for 40 min at a water chiller flow rate of 2.2×10^−5^ m^3^/s (Zhu et al. 2012). A calibration curve was created using different formaldehyde concentrations (0–0.3 μg/mL). A 1 mL acetylacetone solution and 10 mL distillate were mixed and incubated in a boiling water bath for 10 min. At the end of the incubation, the absorbance of the solution was determined spectrophotometrically at 412 nm. Distilled water was used as reference instead of distillate. Measurements and distillation were done separately for each group. Experiments were repeated three times to avoid measurement deviations that may occur due to the heterogeneity of the samples.

### Total soluble protein content

The total soluble protein content of the control and FMD-treated *A. bisporus* samples was analyzed according to the method of Bradford (1976). A 100 mg of the sample and 2 μL of beta-mercaptoethanol were mixed with 20 mL of 01 M pH 7.4 phosphate buffer containing 0.5% polyvinylpyrrolidone, and the samples were homogenized in a precooled pestle and mortar. After homogenization, the mixture was centrifuged at 10,000 rpm for 15 min, and the supernatant was collected for protein determination. A 10 μL of supernatant, 990 μL of phosphate buffer, and 5 mL of dye were mixed and kept in the dark for 5 min. After incubation, the absorbance of the solution was measured spectrophotometrically at 595 nm. The protein content was calculated using the bovine serum albumin calibration curve. Each analysis was repeated three times.

### Total soluble sugar content

For determination of total soluble sugar, samples were extracted with 2 mL of hot 80% ethanol and centrifuged at 10,000 rpm for 20 min. The extraction procedure was repeated twice in the same manner, and the resulting supernatant was collected. The residue obtained by evaporation of the ethanol was dissolved in 5 mL of distilled water. The 0.1 mL samples taken from the extracts were diluted to 1 mL with distilled water, and 4 mL of 0.2% anthrone reagent was added. All mixtures were incubated in a hot boiling water bath for 8 min, and at the end of this time, the absorbance of the solutions was measured at 630 nm. Glucose was used as the standard, and the total amount of soluble sugar was calculated from the calibration curve (Lee et al. 1992).

### Total phenolic and flavonoid content

For the determination of total phenolic content (TPC) and total flavonoid content (TFC), extraction was first performed. And 5 g of dried and grounded mushroom samples were extracted with 70% ethanol by shaking for 5 h at room temperature. At the end of the extraction, the supernatant was filtered after centrifugation at 3000 rpm. The extracts were evaporated to dryness on a rotary evaporator at 40 °C and frozen at -12 °C. The extracts were used for subsequent analysis. Total phenolic content was determined by Folin-Ciocalteu assay, and a mixture containing 1 mL of Folin-Ciocalteu reagent and 3 mL of 20% Na_2_CO_3_ was prepared. A 1 mL of methanol extract was added to mixture, and after incubation in the dark at room temperature for 30 min, the absorbance of the solutions was measured at 765 nm. The results were expressed as mg gallic acid equivalent (GAE) per 100 g dry weight (Ayhan et al. 2019). For TFC determination, 10 mL of methanol extract of the mushrooms and 1 mL of sodium nitrite were mixed and incubated for 6 min. A 1 mL Al(NO_3_)_3_ was added to the mixture and incubated a second time for 6 min, and the total volume was made up to 25 mL with dH_2_O. The absorbance of the final mixture was determined spectrophotometrically at 510 nm after 15 min. of incubation. Quercetin was used as a standard, and flavonoid amounts were expressed in mg/g (Durhan et al. 2022).

### ICP-EOS analysis

Control and FMD-treated *A. bisporus* samples (0.1 g) were digested in a microwave dissolution apparatus for analysis of elements. The combustion process was performed by adding 5 mL of Suprapur nitric acid and 2 mL of hydrogen sulfide to the sample, which was then transferred to the Teflon tubes of the microwave oven. After the combustion process, 50 mL of ultrapure water was added to the sample, and analysis was performed using the ICP-OES instrument (CAP 6500 ICP-OES-TRUE SIMULTANEOUS, Thermo Scientific ICAP QC, USA) (Yıldırım and Apaydın 2021). ICP-MS analysis was carried out by Hitit University-HUBTUAM Center.

### LC-MS/MS analysis

A 2 g of *A. bisporus* samples were extracted with methanol/dichloromethane (4:1) solvent in an ultrasonic bath for 120 min, filtered through a 0.45 μM syringe filter, and analyzed by LC-MS/MS. 0.1% Formic acid-water (A) and methanol (B) were used as solvents. Solvent program was applied as follows: A, 100% for 0–22 min; A, 5% for 22–25 min; and A, 0% for 25–30 min. Flow rate, column furnace temperature, and injection volume were determined as 0.7 mL/min, 30 °C and 20μL, respectively. Octadecylsilane-Hypersil 4.6*250 mm column was used. The MS/MS analysis conditions are as follows: capillary temperature, 300 °C; vaporizer temperature, 350 °C; sheath gas pressure, 30 Arb; Aux gas pressure, 13 Arb; positive polarity, 2500 V; negative polarity, 2500 V; and discharge current, 4 μA (Akman et al. 2023; Kayir et al. 2023). Quantification was evaluated according to MS/MS. The precision, accuracy, and selectivity of the method were evaluated by repeating the measurements at three concentrations for each compound. A good precision was determined, and the method was found to show high selectivity and accuracy. LC-MS/MS analysis was carried out by Hitit University (HUBTUAM).

### Scavenging activity

DPPH radical scavenging activity was calculated according to the method of Gündüz et al. (2021). A 2 mL of DPPH (0.2 mM) and 2 mL of *A. bisporus* samples at different concentrations (0.05–2 mg/mL) were mixed by vigorous shaking. The mixture was allowed to stand at room temperature for 30 min, and at the end of the period, the absorbance of the mixture was measured at 517 nm. H_2_O_2_ scavenging activity was determined according to the method of Keser et al. (2012). A 1 mL of the *A. bisporus* sample at different concentrations (0.05–2 mg/mL) was mixed with 2.4 mL of phosphate buffer and 0.6 mL of H_2_O_2_ solution (40 mM). The mixture was incubated at room temperature for 10 min. Subsequently, the absorbance of the reaction mixture was determined spectrophotometrically at 230 nm. VC was used as a positive control for both scavenging activities. The radical scavenging activity of DPPH and H_2_O_2_ is calculated using the following formula:

$$\textrm{Scavenging}\ \textrm{activity}\ \left(\%\right)=\left(\textrm{A}_0-\textrm{A}_1\right)/\textrm{A}_0\times 100$$

A_0_ is the absorbance of the control, and A_1_ is the absorbance of the sample. The experiment was repeated three times at each concentration.

### In vivo antimutagenic activity

The antimutagenic activity of *A. bisporus* samples was determined using chromosome aberration tests (CAs) in *Allium cepa*. Since the highest change in the content of phytochemicals was observed in the group with 6% FMD application, the change in antimutagenic activity in the control samples and 6% FMD-treated *A. bisporus* was investigated. For this purpose, six different groups of 10 bulbs each were formed, and bulbs were germinated at 24 °C for 72 h, and then chromosomal abnormalities were detected in root tips at the end of germination (Demirtaş et al. 2020). Group I was accepted as a negative control, and the bulbs were germinated with distilled water. In group II, which was determined as a positive control, the bulbs were treated with 25 mg/mL sodium azide (NaN_3_), which is a potent mutagen (Akgeyik et al. 2023). To determine whether the control and 6% FMD-treated samples were mutagenic alone, groups III and IV were germinated with 2 mg/mL control and 2 mg/mL extract from the 6% FMD group, respectively. In groups V and VI, the extract and NaN_3_ were administered together, and antimutagenic activity was determined by the reduction of abnormalities induced by NaN_3_. Group V was germinated with 25 mg/mL NaN_3_ and 2 mg/mL control extract, and group VI was germinated with 25 mg/mL NaN_3_ and 2 mg/mL 6% FMD-treated *A. bisporus* extract CA (%), and antimutagenic activity (%) is calculated using the following equations (Yalçin and Çavuşoğlu 2022):

$$\textrm{CA}\ \left(\%\right)=\textrm{A}/\textrm{B}\ \times100$$

$$\textrm{Antimutagenic}\ \textrm{activity}\ \left(\%\right)=\left[\left(\textrm{a}-\textrm{b}\right)/\left(\textrm{a}-\textrm{c}\right)\right]\ \times\ 100$$

where *A* refers to the cell with total abnormal chromosome, *B* refers to the total counted cell, *a* represents the %CA of the NaN_3_ applied group, *b* represents the %CA of the NaN_3_ and extract applied group, and *c* represents the %CA of the negative control group.

### Statistical analysis

Analyses were performed with the “IBM SPSS Statistics 22” package program, and the data were given as mean standard deviation (SD). Statistical significance between the means was determined by Duncan’s test and one-way ANOVA, and a *p* value of < 0.05 was considered statistically significant.

## Results and discussion

Formaldehyde used in different stages of composting causes changes in the nutritional properties and biological activities of *A. bisporus*. In this study, the effects of formaldehyde (2, 4, and 6%) on total phenolic and flavonoid content, phenolic acid profile, total soluble sugar and protein content, macro- and micro-elements levels, and scavenging and antimutagenic activities of *A. bisporus* were investigated. Firstly, FMD levels in *A. bisporus* samples were determined by the acetylacetone spectrophotometry method. As a result of spectrophotometric analysis, the presence of 2.93 ± 0.29 mg/kg FMD was detected in the control group samples, while the presence of 3.27 ± 0.52 mg/kg, 7.66 ± 0.81 mg/kg, and 11.41 ± 0.93 mg/kg FMD was detected in the 2, 4, and 6% FMD applied groups, respectively. The presence of FMD, even if at a low level, in the control group can be associated with FMD that occurs naturally in *A. bisporus* (Chin and Lindsay 1994). In the 2% FMD applied group, FMD was detected at a dose close to the control group, and in the 4% and 6% FMD applied groups, 2.61 and 3.89 times more FMD were detected, respectively, compared with the control group. The fact that residues were detected at a level close to the control group in the low-dose FMD applied group (2%) can be said that accumulation in the body can be prevented by the detoxification mechanism at this dose and that the detoxification mechanism is insufficient at high doses (4% and 6%). Shao et al. (2023) reported the presence of FMD in the range of 1.29–20.94 mg/kg in *A. bisporus* samples consumed as food in China and purchased from local markets.

### Total soluble sugar and protein content

**Fig. 1:**
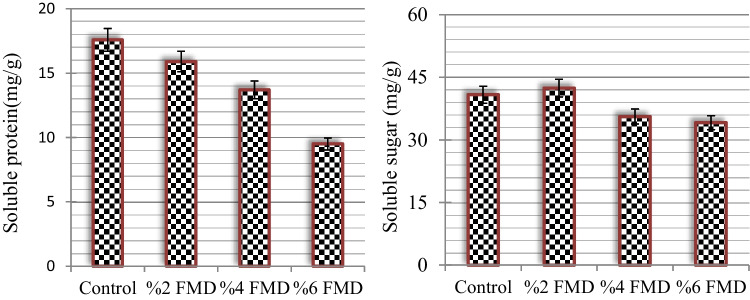
Total soluble sugar and protein content in the control and FMD-treated *A. bisporus*

Edible cultivated mushrooms are a good source of protein and have an important place in nutrition. In the control samples of *A. bisporus*, the soluble protein content was found to be 17.65 ± 0.85 mg/g (Fig. 1). While application of 2% FMD did not cause significant change in protein content (*p* > 0.05), other FMD applications caused significant changes. The most significant change was observed in the group with 6% FMD application; soluble protein content decreased by 46% to 9.52 ± 0.25 mg/g in this group. These results show that high-dose FMD application causes a reduction in the nutritional value of *A. bisporus*. The protein content of *A. bisporus* samples grown in different environments has been studied in the literature, but there is no study reporting the effects of FMD. Nissa et al. (2022) reported a protein content of 12.86 ± 0.23 mg/g in *A. bisporus* samples from India and noted that mushrooms are a good source of protein. Sadiq et al. (2008) reported a protein content of 11.01 % in *A. bisporus* samples grown in Iran. The total amounts of soluble sugar in the control and FMD-treated *A. bisporus* samples are shown in Fig. 1. A 40.83 mg/g total sugar content was detected in the control group samples. In the 2% FMD treatment, the total sugar content was 42.47 mg/g, which was not a statistically significant difference from the control group (*p* > 0.05). In contrast to the 2% FMD application, the total sugar level was found to decrease in the 4% and 6% FMD applications. A significant difference in sugar content was found in 4% and 6% FMD applications compared with the control group (*p* < 0.05), but no statistical difference was found between the %4 FMD and %6 FMD groups (*p* > 0.05). Total sugar content, which was 35.6 mg/g in the group with 4% FMD application, was determined to be 34.12 mg/g in the group with 6% FMD application. The decrease in sugar levels, especially as a result of the application of 6% FMD, also affects many biochemical metabolic pathways. Carbohydrates are the precursors of phenolic substances and phytoalexins, and disruption of carbohydrate metabolism also affects the pathway of important secondary compounds. This claim is also supported by the results of LC-MS/MS analysis. In the literature, the sugar content of *A. bisporus* samples grown under different climatic conditions was studied, but there is no data on the effects of FMD. Nissa et al. (2022) reported the presence of 50.53 ± 0.16 mg/g sugar in *A. bisporus* samples grown in Jammu (India).

### Total TPC and TFC levels

**Fig. 2:**
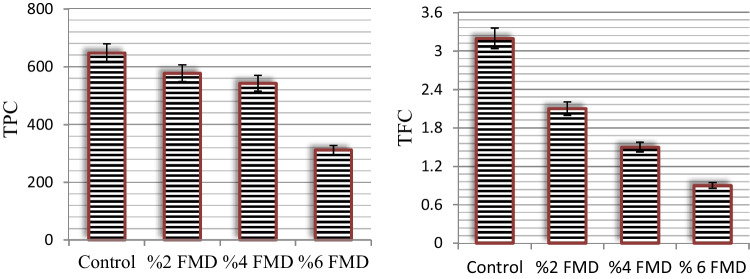
The TPC (mgGAE/100 g) and TFC (mg/g) in the control and FMD-treated *A. bisporus*

The TPC and TFC in the control and FMD-treated *A. bisporus* samples are shown in Fig. 2. After the application of FMD, a decrease in TPC was observed in the control *A. bisporus* samples, with 648.2 ± 12.3 mg GAE/100 g level. In samples treated with 2, 4, and 6% FMD, the TPC content decreased by 1.12, 1.19, and 2.07 times, respectively. This decrease was similar in the groups with 2% and 4% FMD application, and the most significant decrease was observed in the group with 6% FMD application. The decrease in TPC was also found to be dependent on the dose of FMD applied, with the most significant decrease determined in the group with 6% FMD application. FMD application decreased the TFC levels by 34.4–71.8% compared with the control group. These decreases indicate that FMD application leads to disruption of many metabolic processes in *A. bisporus*. Although there are studies in the literature that examine the phenolic and flavonoid content of *A. bisporus*, there is no study that examines the effect of FMD applied during the cultivation phase. Gąsecka et al. (2018) reported 132.7–542.7 mg GAE/100 g TPC content in differentially cultured *A. bisporus* samples. Palacios et al. (2011) reported TFC content of about 1.1 mg/g in cultured *A. bisporus* samples. The TPC and TFC values obtained in this study are compatible with the literature. As a result of FMD application, the decreases in phenolic content in particular indicate a loss of antioxidant activity. To confirm the decrease in phenolics in the control and FMD application groups, LC-MS/MS analysis was performed and the profile of phenolic compounds of the samples from the control group with 6% FMD-applied *A. bisporus*, where the highest loss was observed, was examined.

### LC-MS/MS analysis

**Fig. 3:**
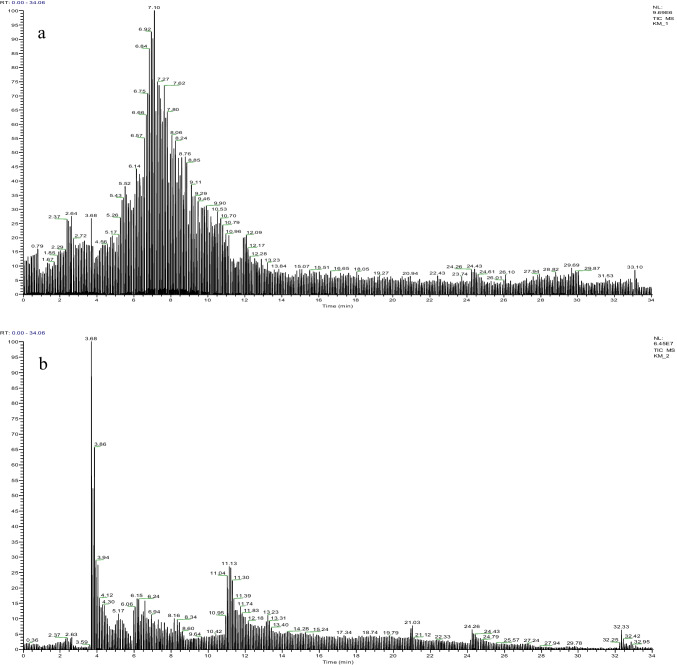
LC-MS/MS chromatograms. a: control, b: formaldehyde-treated *A. bisporus*

The profile of phenolic substances and the presence rates of the control and 6% formaldehyde-treated *A. bisporus* are shown in Table 1. LC-MS/MS chromatograms and calibration equations are given in Fig. 3 and Table 2, respectively. A total of 29 standard compounds were tested. While five of the standards were detected in the control group, four of them were detected in the 6% FMD applied group. Only the content of quercetin is similar in both samples, and the amounts of quercetin were determined in similar amounts in both samples. Protocatechuic acid (15.046 μg/g), which was the highest detected in the control-*A. bisporus*, was not detected in the 6% formaldehyde treated sample. Salicylic acid, ferulic acid, and 4-hydroxy benzoic acid (4-HBA), which were detected in the control, were also not detected in the formaldehyde-treated sample. Phenolic content was found to be significantly weakened in the formaldehyde-treated sample, with lower levels of rosmarinic acid, oleuropein, and resveratrol. It has been reported in the literature that *A. bisporus* contains different types and ratios of phenolic compounds. Palacios et al. (2011) reported that *A. bisporus* contains caffeic acid, chlorogenic acid, p-coumaric acid, ferulic acid, gallic acid, p-hydroxybenzoic acid, and protocatechuic acid, as well as homogentistic acid, myricetin, and pyrogallol. Kim et al. (2008) quantified gallic acid, protocatechuic acid, myricetin, and pyrogallol in *A. bisporus*. The differences found in this study compared to literature studies investigating the profile of phenolic compounds of *A. bisporus* may be related to the different growing conditions, compost structure, and climatic conditions (Durhan et al. 2022). In contrast to literature studies, the formaldehyde used in the cultivation phases was found to cause a change in phenolic content in this study. Formaldehyde occurs naturally in many organisms and mushroom. Formaldehyde produced in the mushroom plays an important role in chemical defense against infecting microorganisms. Formaldehyde, formed slowly during the growth and development process, is produced from lentic acid and has a direct fungicidal effect against mycoparasites (Yasumoto et al. 1971; Himstedt et al. 2020). The increase in formaldehyde concentration in mushroom tissues as a result of exposure to in vivo production can lead to interruptions in many biochemical reactions. Formaldehyde reacts with proteinogenic and nucleophilic amino acids to form cyclized, hydroxymethylated, N-methylated, and N-formylated products. These reactions result from the cross-linking of the amino group of formaldehyde with amino, imino, amide, sulfhydryl, and hydroxyl groups in amino acids. In this way, formaldehyde can cause abnormalities and loss of function in many protein and enzyme structures (Kamps et al. 2019). The decrease in phenolic compounds in the *A. bisporus* samples treated with formaldehyde can be explained by the possible denaturation of the enzymes involved in the synthesis reactions. Another reason for the decrease in phenolic compounds could be that formaldehyde, a reactive carbonyl compound, reacts readily with phenolic compounds at low temperatures (40–50 °C). The high reactivity of phenols with formaldehyde suggests the use of polyphenols in the scavenging process of this compound (Li et al. 2023). Polyphenols are important antioxidant compounds and provide important defense against cell damage (Macar et al. 2020; Aydin et al. 2022; Kurt et al. 2023). Takagaki et al. (2000) reported that some polyphenols such as epigallocatechin and epigallocatechin gallate eliminated formaldehyde at a rate of 86.4% and 81.5%, respectively. The involvement of phenolic substances in the elimination of formaldehyde also leads to a decrease in the content of these active compounds. The decrease in the content of phenolic substances observed in this study could be related to a delay/pause in the synthesis steps or scavenging activity. As a result, the presence of phenolic content in the *A. bisporus* samples of both groups ensures that the cultivated mushroom exhibits antioxidant activity. Consuming foods with antioxidant activities also reduces the risk of many diseases by reducing the effects of free radicals (Yalçın and Çavuşoğlu 2022; Üstündağ et al. 2023; Çavuşoğlu and Yalçın 2023).

**Table 1:**
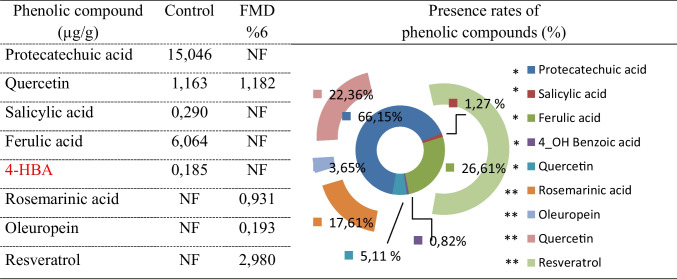
Phenolic profile and the presence rates in control-*A. bisporus* and 6% formaldehyde-treated *A. bisporus*

*represents control group data, ** represents 6% FMD group data. Phenolic compounds are given as μg/g. *NF* not found

**Table 2:** *R*^2^ and calibration equations of standard phenolic compounds

Phenolic	Equation	*R*^2^	Linear range*
Pyrogallol	y = 460246x	0.9982	0.125–4 ppm
Gallic acid	y = 177194+930869x	0.9850	0.125–4 ppm
Protocatechuic acid	y = -25814.7+575164x	0.9983	0.125–4 ppm
Protocatechuic aldehyde	y = 2.30978e+006+8.03118e+006x	0.9984	0.125–4 ppm
Sesamol	y = 837472+5.4916e+006x	0.9961	0.125–4 ppm
Catechin	y = -904664+5.03151e+007x	0.9999	0.125–4 ppm
Gentisic acid	y = 3.25247e+006x	0.9985	0.125–4 ppm
Epicatechin	y = 1.44722e+006+1.19049e+008x	0.9988	0.125–4 ppm
Caffeic acid	y = 489708+6.15728e+006x	0.9991	0.125–4 ppm
Vanillin	y = 43751.4+2.51555e+006x	0.9994	0.125–4 ppm
Syringic acid	y = 1.42128e+007+3.06105e+008x	0.9987	0.125–4 ppm
Syringic aldehyde	y = 8.49417e+006x	0.9989	0,125–4 ppm
Taxifolin	y = 31342.9+5.00026e+006x	0.9994	0.125–4 ppm
p_coumaric acid	y = 226019+866981x	0.9985	0.125–4 ppm
sinapic_acid	y = 41331.9x	0.9952	0.125–4 ppm
Salicylic acid	y = 17336.1+4.50111e+007x	0.9989	0.125–4 ppm
Ferulic acid	y = -6835.99+1.10517e+006x	0.9985	0.125–4 ppm
4-HBA	y = -43272.1+4.05244e+007x	0.9993	0.125–4 ppm
Rosemarinic acid	y = -336870+8.4937e+006x	0.9999	0.125–4 ppm
Oleuropein	y = -62301.3+9.74238e+006x	0.9991	0.125–4 ppm
Routine	y = -1.27749e+006+5.94364e+007x	0.9950	0.125–4 ppm
Resveratrol	y = 2.0925e+006+9.92589e+006x	0.9986	0.125–4 ppm
Ellagic acid	y = -162221+1.22026e+007x	0.9962	0.125–4 ppm
Naringenin	y = 853007x	0.9954	0.125–4 ppm
Salicylic acid	y = 1.79711e+008x	0.9941	0.125–4 ppm
Quercetin	y = -2.11562e+006+3.71335e+007x	0.9983	0.125–4 ppm
Kaempferol	y = 9.26487e+006+2.271e+007x	0.9948	0.125–4 ppm
Galangin	y = 1.26793e+007x	0.9954	0.125–4 ppm
Flavone	y = 4.10946e+007+6.9719e+008x	0.9995	0.125–4 ppm

*Linear range represents the calibration range studied for standard compounds. Standards were used between the smallest value (0.125 ppm) and the largest value (4 ppm)

### Scavenging activity

**Fig. 4:**
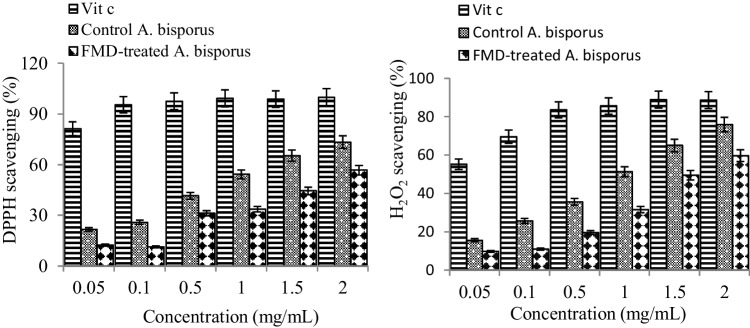
DPPH and H_2_O_2_ scavenging activities of vitamin C, control and 6% FMD-treated *A. bisporus*

The DPPH scavenging activities of vitamin C, control, and the FMD-treated *A. bisporus* are shown in Fig. 4. The DPPH scavenging activity of both *A. bisporus* samples increased in a dose-dependent manner. At concentrations of 0.05–2 mg/mL, the standard compound, vitamin C, exhibited H_2_O_2_ scavenging activity in the range of 55.2–88.7%, control *A. bisporus* exhibited activity in the range of 15.6–75.9%, and 6% FMD-treated *A. bisporus* exhibited activity in the range of 9.7–59.7%. FMD application reduced H_2_O_2_ scavenging activity, and a similar decrease was also observed in DPPH scavenging activity. At concentrations of 0.05–2 mg/mL, the control *A. bisporus* samples showed DPPH scavenging activity in the range of 21.6–73.3%, while the 6% FMD-treated *A. bisporus* samples showed activity in the range of 12.3–56.7% at the same concentrations. The highest activity of both samples was obtained at a dose of 2 mg/mL, and application of FMD at this dose reduced the DPPH scavenging activity by 22.6%. Formaldehyde can cause the production, accumulation of reactive oxygen species, and oxidative stress in cells and tissues. Oxidative stress causes damages by attacking many macromolecules (Macar et al. 2023; Himtaş et al. 2024; Coşkun et al. 2024). Phenolic compounds prevent cell damage by reducing oxidative stress (Yılmaz et al. 2023). In this study, *A. bisporus* was found to have DPPH radical scavenging activity and to be an antioxidant, but this activity decreased after FMD exposure. This decrease is directly related to the decrease in phenolic content as determined by LC-MS/MS analysis. Protocatechuic acid, which was detected in the control samples but not in the FMD-treated samples, is a potent antioxidant. It exhibits this property by inhibiting the formation of free radicals, preventing the effects of free radicals, and acting as a metal chelator. In addition to the antioxidant effect of protocatechuic acid, its anti-microbial, anti-diabetic, anti-ulcer, anti-viral and anti-inflammatory effects have also been reported in the literature (Akgeyik et al. 2023). Quercetin, which like protocatechuic acid is undetectable in FMD-treated mushrooms, acts as a potent antioxidant substance by directly scavenging free radicals. This strong antioxidant property of quercetin is due to the catechin group in the molecule and especially to the -OH group. The strong antioxidant quercetin suppresses oxidative stress and protects cells from damage (Mohammed et al. 2021; Onur et al. 2023).

### ICP-EOS analysis

The levels of macro- and microelements in the control and 6% FMD- treated *A. bisporus* are shown in Table 3. The macroelements detected in high concentrations in the control *A. bisporus* were K (13291.500 ppm), Ca (492.300 ppm), Mg (459.600 ppm), and Na (315.300 ppm). FMD application resulted in an increase in some elements and a decrease in others. While the elements that increase after 6%-FMD application are Ag, Al, B, Ca, K, and Mg, the elements that decrease are Cd, Co, Cr, Cu, Fe, Na, Mn, and Zn. Among the elements, the most significant decrease was observed in Cr and Cd, and FMD application reduced the values of these elements by 82.6% and 70.1%, respectively. Among the elements, the most significant increase was observed in B and Al, and 6% FMD application increased the values of these elements by 23.63% and 16.9%, respectively. These decreases and increases may be associated with anomalies in the supply of elements from the environment after FMD application. Although there are studies in the literature that examine the content of elements in *A. bisporus* samples, there is no study that examines the effects of FMD on these elements. Krishnamoorthi et al. (2022) reported the presence of Mg (115.05 mg/100 mg), Mn (0.87 mg/100 mg), P (1230.63 mg/100 mg), Na (28.76 mg/100 mg), Ca ( 107.21 mg/100 mg), and K (2925.14 mg/100 mg) in *A. bisporus* samples grown in Tamil Nadu (India).

**Table 3:**
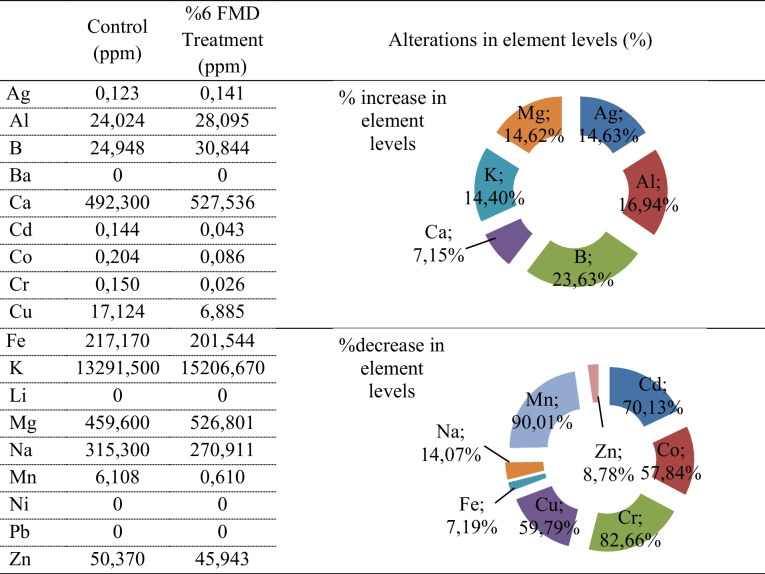
The levels of macro- and microelements in the control and 6% FMD-treated *A. bisporus*

### Antimutagenic activity

**Fig. 5:**
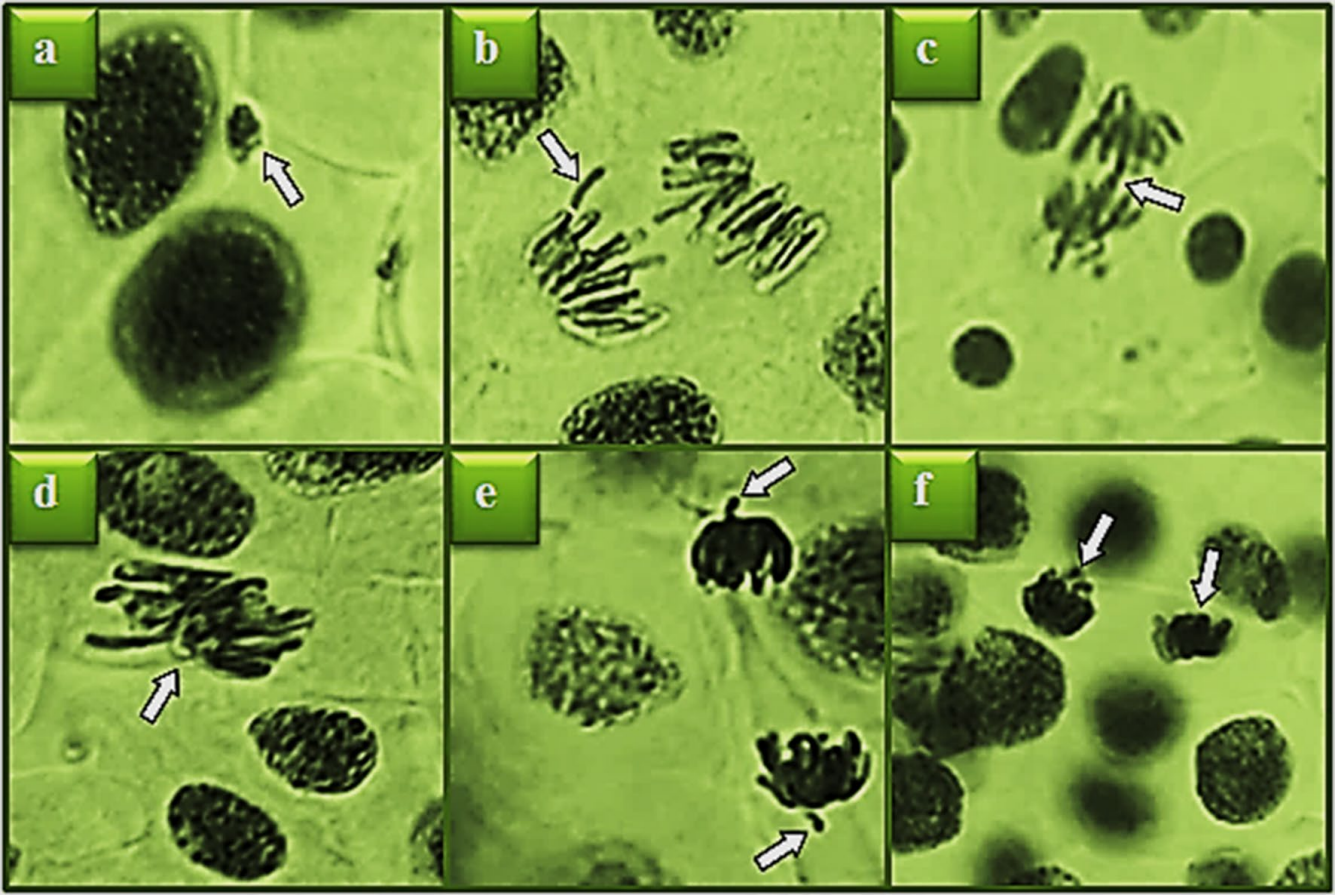
CAs induced by NaN_3._ MN (**a**), fragment (**b**), bridge (**c**), sticky chromosome (**d**), vagrant chromosome (**e**), and reverse polarization (**f**)

The antimutagenic activity of the control and the %6 FMD-treated extract are shown in Table 4. A non-statistically significant incidence of CA was observed in the negative control group and in the groups receiving only *A. bisporus* extracts. This result indicates that *A. bisporus* alone has no genotoxic effect. The high frequency of abnormalities in the positive control group indicates the mutagenic effect of NaN_3_ (Fig. 5). Among the abnormalities, MN formations were detected at the highest rate. This abnormality, which is observed with high frequency, may be caused by anogenic or clastogenic effects in the cells that cause spindle fiber damage, chromosome breaks, stuck chromosomes, or reverse polarization. All these toxic effects confirm that NaN_3_ is a positive mutagen, so NaN_3_ is preferred positive control in antimutagenicity studies (Kuloğlu et al. 2022; Güç et al. 2022). The antimutagenic activity of the *A. bisporus* samples was determined considering the decrease in the abundance of MNs and CAs induced by NaN_3_. Both the control and FMD-treated *A. bisporus* extracts showed antimutagenic activity by decreasing the frequency of MNs and CAs induced by NaN_3_. The control *A. bisporus* sample showed high antimutagenic activity and provided over 40% antimutagenic protection against all abnormalities. Antimutagenic activity of the %6 FMD-treated *A. bisporus* extract was reduced compared to the control group. While the extract of the control showed antimutagenic activity in the range of 40.4–95.5%, the 6% FMD-treated *A. bisporus* extract showed activity in the range of 20.8–47.1%. The highest antimutagenic activity in the control *A. bisporus* extract was determined to be 95.5% against the sticky chromosome. In the %6 FMD-treated *A. bisporus* extract, this protection decreased to 33.9%. While the control extract exhibited high antimutagenic activity against sticky chromosome, the 6% FMD-treated *A. bisporus* extract almost completely lost its antimutagenic activity against this abnormality. The antimutagenic activity of *A. bisporus* can be explained by the active phenolic substances it contains. Protocatechuic acid, the major phenolic compound found in *A. bisporus* control samples, is a potent antimutagen, and this effect is due to its anti-oxidant property. Protocatechuic acid has an antioxidant effect by preventing the production of free radicals and scavenging free radicals in the cell. This effect also provides antimutagenic activity (Dare et al. 2020). The presence of protocatechuic acid, which was highly detected in the control *A. bisporus* by LC-MS/MS analysis, was not found in the 6% FMD-treated samples. Ferulic acid, the second major compound detected in control *A. bisporus*, also exhibits antimutagenic activity through several mechanisms. Yamada and Tomita (1996) reported that ferulic acid significantly reduced mutagen-induced mutagenicity in the *Salmonella typhimurium* system. In this study, the change observed in protocatechuic acid after FMD application was also observed in ferulic acid. While 6.064 μg/g ferulic acid was detected in the control sample, it was not detected in the FMD-treated samples. Thus, the decrease in the antimutagenic activity of the samples treated with FMD can be explained by the serious decrease in the content of phenolic substances.

**Table 4:** The effects of FMD application on anti-mutagenic activity of *A. bisporus*

	Negative control	Positive control	Control *A. bisporus*	%6 FMD treated *A. bisporus*	NaN_3_+Control *A. bisporus*	NaN_3_+%6 FMD treated *A. bisporus*
MN	0.00±0.00^d^	111.8±3.9^a^	0.19±0.15^d^	0.36±0.19^d^	65.6±4.1^c^	85.9±4.2^b^
F	0.00±0.00^d^	93.7±6.3^a^	0.00±0.00^d^	0.11±0.05^d^	55.8±3.9^c^	71.4±2.7^b^
B	0.13±0.09^d^	75.4±6.3^a^	0.00±0.00^d^	0.00±0.00^d^	31.4±2.9^c^	59.7±3.1^b^
RP	0.00±0.00^d^	51.2±4.5^a^	0.00±0.00^d^	0.00±0.00^d^	23.6±3.1^c^	33.8±4.9^b^
VC	0.00±0.00^d^	31.9±1.7^a^	0.24±0.11^d^	0.39±0.14^d^	12.6±1.6^c^	21.5±2.3^b^
SC	0.21±0.11^c^	22.3±2.2^a^	0.00±0.00^c^	0.44±0.20^c^	0.99±0.13^c^	21.9±1.1^b^

Means shown with different letters^(a–d)^ on the same line are statistically significant (*p* < 0.05). *MN* micronucleus, *F* fragment, *B* bridge, *RP* reverse polarization, *VC* vagrant chromosome, *SC* sticky chromosome

## Conclusion

FMD used for disinfection at different stages of *A. bisporus* cultivation caused significant losses in phytochemical content and biological activity. FMD application caused a decrease in the nutritional value of *A. bisporus*, such as total soluble sugar and protein, TPC, and TFC content, as a function of increasing dose. Protocatechuic acid, salicylic acid, ferulic acid, and 4-hydroxy benzoic acid, which were detected in the control group by LC-MS/MS analysis, were not detected in the 6% FMD applied group. Phenolic compounds have an important place in the emergence of many biological activities. The decrease in phytochemical content in the FMD applied group also caused a decrease in important biological activities. The decrease in phenolic compounds with high antioxidant activity also reduced the DPPH scavenging activity of *A. bisporus*. Antimutagenic activity decreased along with the loss of antioxidant activity after exposure to FMD. *A. bisporus*, which has antimutagenic activity in the range of 40.4–95.5%, showed antimutagenic activity in the range of 20.8–47.1% after 6% FMD exposure. This study, which shows that FMD contamination causes negative effects on the nutritional value and biological activities of *A. bisporus*, will guide the planning of future studies. It is also very important to investigate the toxic effects and carcinogenic activity of *A. bisporus* contaminated with FMD on mammals. It is recommended to develop natural alternative natural agents and methods instead of formaldehyde used in various stages of mushroom cultivation. In particular, the effectiveness of natural herbal extracts, essential oils, and secondary metabolites against fungal pathogens can be tested and used instead of chemical disinfectants.

## Data Availability

All data generated or analyzed during this study are included in this article.

## Abbreviations

4-HBA: 4_ OH benzoic acid
CAs: Chromosomal abnormalities
DPPH: 2,2-Diphenyl-1-picrylhydrazyl
FMD: Formaldehyde
ICP-EOS: Inductively coupled plasma optical emission spectrometry
LC-MS/MS: Liquid chromatography mass spectrometer
TFC: Total flavonoid content
TPC: Total phenolic content
